# Intranasal vaccine from whole *Leishmania donovani* antigens provides protection and induces specific immune response against visceral leishmaniasis

**DOI:** 10.1371/journal.pntd.0009627

**Published:** 2021-08-17

**Authors:** Doumet Georges Helou, Aurélie Mauras, François Fasquelle, Juliane Sousa Lanza, Philippe M. Loiseau, Didier Betbeder, Sandrine Cojean

**Affiliations:** 1 Université Paris-Saclay, CNRS, BioCis-UMR 8076, Châtenay-Malabry, France; 2 Université de Lille, Inserm, LIRIC-UMR 995, Lille, France; Federal University of Rio de Janeiro, BRAZIL

## Abstract

Visceral leishmaniasis is a protozoan disease associated with high fatality rate in developing countries. Although the drug pipeline is constantly improving, available treatments are costly and live-threatening side effects are not uncommon. Moreover, an approved vaccine against human leishmaniasis does not exist yet. Using whole antigens from *Leishmania donovani* promastigotes (LdAg), we investigated the protective potential of a novel adjuvant-free vaccine strategy. Immunization of mice with LdAg *via* the intradermal or the intranasal route prior to infection decreases the parasitic burden in primary affected internal organs, including the liver, spleen, and bone marrow. Interestingly, the intranasal route is more efficient than the intradermal route, leading to better parasite clearance and remarkable induction of adaptive immune cells, notably the helper and cytotoxic T cells. *In vitro* restimulation experiments with *Leishmania* antigens led to significant IFN-γ secretion by splenocytes; therefore, exemplifying specificity of the adaptive immune response. To improve mucosal delivery and the immunogenic aspects of our vaccine strategy, we used polysaccharide-based nanoparticles (NP) that carry the antigens. The NP-LdAg formulation is remarkably taken up by dendritic cells and induces their maturation *in vitro*, as revealed by the increased expression of CD80, CD86 and MHC II. Intranasal immunization with NP-LdAg does not improve the parasite clearance in our experimental timeline; however, it does increase the percentage of effector and memory T helper cells in the spleen, suggesting a potential induction of long-term memory. Altogether, this study provides a simple and cost-effective vaccine strategy against visceral leishmaniasis based on LdAg administration *via* the intranasal route, which could be applicable to other parasitic diseases.

## Author summary

Visceral leishmaniasis is a neglected tropical disease caused by specific species of *Leishmania* parasites that affect internal organs including spleen, liver, and bone marrow. The infective stage called promastigote, is transmitted into the host skin *via* sandfly bites. Visceral leishmaniasis is usually associated with high mortality rate in poor and developing countries, lacking proper health assistance. Moreover, treatments are expensive while no approved vaccines exist to prevent infection and avoid disease outbreaks. This study suggests an affordable and adjuvant-free vaccine formulation made from the total lysate of promastigotes. Vaccine administration *via* the intranasal route, ensures a remarkable clearance of *Leishmania* parasites from the internal organs of infected experimental mice. In particular, intranasal route known to be not invasive, is efficient in inducing adequate immune response against the infective form of the parasite. Further studies are now required to improve this prophylactic vaccine and provide therefore the basis for a promising translational approach.

## Introduction

Visceral leishmaniasis (VL) is a parasitic disease that could be fatal in the absence of appropriate medical treatment. According to the World Health Organization, outbreaks and re-emergences were reported in 83 countries in 2018, including east Africa, India, Bangladesh and Brazil [1]. *Leishmania donovani* and *Leishmania infantum* are the causative agents, with female phlebotomine sandflies considered to be the principal vectors. The promastigote flagellar form of the parasite is inoculated into the skin during a blood meal. Once in the dermis of the host, the parasite primarily infects antigen-presenting resident cells, including dendritic cells (DCs), and transforms within macrophages into a proliferative aflagellar form, known as amastigote. This promotes the dissemination of the parasite *via* the vascular and lymphatic systems, leading to the infiltration of the bone marrow (BM), liver, spleen and several lymph nodes [2,3]. Although treatments against VL exist, their use is limited by adverse effects, emerging resistance and unaffordability in developing countries. Therefore, there is an urgent need for effective vaccines that sufficiently controls leishmaniasis and decreases the leishmaniasis-associated death toll.

*Leishmania* is an opportunistic parasite that highjacks the weakened immune system of vulnerable individuals. In contrast, adapted antileishmanial immunity is the frontline response against *Leishmania* in immunocompetent individuals [4–6]. First, classical activation of infected macrophages promotes their oxidative burst that is associated with superoxide production, leading to the elimination of intracellular parasites [7–9]. DC-*Leishmania* interactions are also essential in driving adaptive immunity towards the activation of T helper 1 (Th1) cell subtype to the detriment of Th2 phenotype. Failure to induce a sustained Th1 response with elevated levels of interferon gamma (IFN-γ) alters the control of Leishmaniasis infection and leads to progressive immune tolerance. The resulting tolerogenic microenvironment drives the alternate activation of macrophages; therefore, enhancing the intracellular proliferation and dissemination of the parasite [10–13]. Although the Th1/Th2 paradigm may shape disease progression, host-*Leishmania* interactions are very complex and implicates other immune players such as Th17 and neutrophils. In particular, IL-17 secretion correlates with better recovery from VL in humans and protects against re-exposure to the parasite [14,15]. Thus, a successful vaccine against *Leishmania* should ensure long-term memory with preferential Th1/Th17 immune response.

Although there are no approved vaccines against VL in humans, several candidates have been developed and tested over the last decades, ranging from inactivated or live attenuated *Leishmania* parasite (first generation) to recombinant *Leishmania* antigens (second generation) and deoxyribonucleic acid (DNA)-based vaccines (third generation) [4,16]. The common limitation of these strategies is the identification of immunogen-specific antigens that are able to elicit appropriate cellular immune responses. While first generation vaccines may be associated with toxicity, they are advantageous with regard to their ability to largely mimic the natural infection and are cost-effective compared to the other generations. To improve the efficacy of first generation vaccines, recent studies have suggested the use of radio-attenuated parasites, the addition of potent adjuvants or a shift towards the unconventional route of vaccination, particularly the elicitation of mucosal immunity [16–22].

In this study, we established a VL mouse model using clinical isolates of *L*. *donovani* to evaluate the efficacy of whole *Leishmania donovani* promastigote antigens (LdAg) from *L*. *donovani*. Through head-to-head comparison, we demonstrated that vaccine administration *via* the intranasal (IN) route induces a complex and specific immune response. We then used a delivery system based on maltodextrin nanoparticles (NP) to improve the uptake and immunogenicity of our LdAg-based vaccine. Altogether, this work highlights the dual efficacy of the IN route and LdAg in the development of a new vaccine against VL.

## Material and methods

### Ethics statements

All animal studies were performed according to European Commission guidelines in compliance with French Animal Welfare Law (law n°2013–1118 from February 1st 2013, article R214.89). Experimentation protocols were approved by the institutional ethic committee for the handling of animals at Paris-Saclay University (CEEA 26-063/2013).

### Mice and parasites

Female BALB/c mice were purchased from Janvier Lab and handled in accordance with the principles and procedures outlined in Council Directive 2010/63/EU. Age- and sex-matched mice were vaccinated at 8–10 weeks of age.

*Leishmania donovani* (MHOM/ET/67/HU3, known as LV9) promastigotes, were cultured in M199 medium (Gibco, Invitrogen) supplemented with 0.1 mM adenosine, 5 μg/mL hemin, 25 mM 4-(2-hydroxyethyl)-1-piperazineethanesulfonic acid (HEPES), 25 mM NaHCO_3_ (Sigma-Aldrich), and 10% heat-inactivated fetal bovine serum (FBS) (Gibco, Invitrogen). Promastigotes were maintained at 25°C, neutral pH, in a dark environment under an atmosphere of 5% CO_2_ [23,24]. For infection, mice were injected with 10^3^ promastigotes at the stationary phase of growth, *via* the intradermal (ID) route [25,26]. All mice groups were infected in our *in vivo* study.

### LdAg preparation and vaccination

For LdAg preparation, promastigotes were accordingly washed with PBS to eliminate the culture medium. Ten cycles of freezing at -80°C, centrifugation and thawing at 37°C, followed by 10 cycles of sonication were necessary to obtain a soluble antigenic mixture. LdAg protein concentration was then determined using microBCA assay (Pierce). To obtain maltodextrin-based nanoparticle (NP)-LdAg formulation, LdAg were mixed with NP at a 1:3 weight ratio (for example 15 μg LdAg with 45 μg NP), in water and at room temperature. The complete antigen encapsulation was confirmed by loading the mixture onto native polyacrylamide gels electrophoresis, followed by a silver nitrate staining **(S1 Table and S1 Fig)**, as previously described [27].

For vaccination, mice were injected at day 0 with 15 μg of LdAg or NP-LdAg, either by the ID route (50 μl) or the IN route (20 μL). At day 21, mice received a booster dose (15 μg) of LdAg or NP-LdAg *via* the same route as the prime dose. All mice groups were infected at day 35.

### Generation and stimulation of bone marrow derived DCs (BMDCs)

Cells were collected from the femur and tibia and resuspended in PBS solution containing 0.5% bovine serum albumin (BSA) and 2 mM Ethylene diamine tetraacetic acid (EDTA) [28,29]. Collected cells were filtrated through 70 μM pre-separation filters to remove cell aggregates or large particles. 3 x 10^6^ viable cells were then cultured (37°C, 5% CO2) in complete Iscove’s Modified Dulbecco’s Medium (IMDM, Gibco, Invitrogen) containing 5% FBS, 1% Penicillin-Streptomycin, 0.4% β-mercaptoethanol (Sigma Aldrich) and granulocyte-macrophage colony-stimulating factor (GM-CSF) at 25 ng/ml (Miltenyi Biotec). Non-adherent cells were seeded in new dishes and enriched with the same medium at days 3 and 6. At day 9, non-adherent cells were recovered, washed and resuspended in supplemented IMDM. For *in vitro* stimulation, collected BMDCs were incubated with 3 μg of LdAg, NP-LdAg or 1 μg/ml Lipopolysaccharide (LPS, ThermoFisher). Purity was checked by flow cytometry and was ≥90%. The uptake of NP and NP-LdAg was also assessed by flow cytometry. Briefly, BMDCs were incubated with increasing amounts of NP-FITC or NP-LdAg-FITC (1% FITC w/w) for 24 h and endocytosis was evaluated using the Attune NxT (ThermoFisher).

### Antibodies and flow cytometry

The following antibodies were used to characterize BMDCs and assess their activation state: MHC II-PE, CD11c-APC, CD86-PE-Cy7, CD80-FITC and CD11b-APC-Cy7 (all from BioLegend). Acquisition was performed on an Attune NxT (ThermoFisher). Leukocyte populations from BM, liver and spleen were also analyzed by flow cytometry. Single cells were excluded from dead cells using the LIVE/ DEAD Zombie NIR Fixable Viability Kit (BioLegend). Immuno-phenotyping was performed using the following antibodies: CD45-BV510, CD49b-PE-Dazzle, CD19-PerCP, CD3-FITC, CD4-AF700, CD8-BV785, C44-BV650 and CD62L-BV421 (all from BioLegend). Full minus one (FMO) controls were used to determine positivity. Precision count beads (BioLegend) were used to count immune cells in different organs. Before acquisition, stained cells were fixed with 1% Paraformaldehyde (Sigma-Aldrich). Acquisition was performed using the BD LSRFortessa and data were analyzed using the FlowJo software (TreeStar) version 10.

### Real Time PCR (qPCR) analysis

Spleen, liver and BM were collected from experimental mice and processed on 70 μM cell strainers to obtain single cell suspensions. qPCR was performed with a total amount of 75 ng of genomic DNA (gDNA). Briefly, gDNA was extracted using DNA extraction kit (Bioline, Meridian Bioscience) according to the manufacturer’s protocol. The parasitic burden was assessed through the amplification of *cytochrome c* gene using the following primers (forward: [5’-CCTGCTCCTCTCCACACA-3’]; reverse [5’-TTCCTCACTCTCCGCTTCTC-5’]). The amplifications cycles were applied as following: 94°C for 7 min, followed by 35 cycles at 94°C for 35 s, 60°C for 35 s, and 72°C for 35 s. At the end of each run, a melting curve analysis was performed from 55°C to 95°C to monitor primer dimers and verify amplicon specificity. The reactions were performed in triplicates. gDNA isolated from *L*. *donovani* promastigotes was used to establish the quantification standard and the gDNA of mouse macrophage RAW 264.7 as negative control. We considered 75 ng of leishmanial DNA to be equivalent to 7.5 x 10^5^ parasites based on the conversion between the quantification of leishmanial DNA and parasites.

### Culture and *in vitro* stimulation of splenocytes

Spleens were collected from infected mice and processed as described above to obtain a single cell suspension. Red blood cells were lysed using commercial buffer (BioLegend) per the manufacturer’s instructions. Cells were resuspended in complete Gibco Dulbecco’s Modified Eagle Medium (DMEM, Gibco, Invitrogen) supplemented with 10% FBS, 10 mM HEPES and 50 μM β-mercaptoethanol, then plated at 2 × 10^5^ cells/well and incubated with or without 3 μg of LdAg. Concanavalin A (Con A, ThermoFisher) was used at 2.5 μg/ml to make positive controls. Supernatants were collected after 72 hours and analyzed using the ELISA Max Deluxe Set Mouse IFN-γ from BioLegend.

### NP preparation

Maltodextrin was dissolved in 2 N sodium hydroxide with magnetic stirring at room temperature. Reticulation and cationization were performed using epichlorohydrin and glycidyl trimethyl ammonium chloride (Sigma-Aldrich). Obtained hydrogels were neutralized with acetic acid and sheared using a high-pressure homogenizer (LM20, Microfluidics, France). The resulting nanoparticles were purified in ultrapure water by tangential flow ultrafiltration using a 750 kDa membrane (AKTA flux 6, GE Healthcare, France), then mixed with 1,2-dipalmitoyl-*sn*-glycero-3-phosphatidylglycerol above the gel-to-liquid phase transition temperature. The average size and zeta potential of maltodextrin nanoparticles were measured in water with the zetasizer nanoZS (Malvern Instruments) by dynamic light scattering and by electrophoretic mobility analysis, respectively **(S1 Table)**. The association of LdAg with NP was characterized using native polyacrylamide gel electrophoresis (native PAGE) **(S1 Fig)**[27]. In some conditions, nanoparticles were conjugated to 1% fluorescein isothiocyanate (FITC, w/w ratio) to assess the uptake of nanoparticles by BMDCs *in vitro*, using flow cytometry.

### *In situ* cytokine quantification

Half of the spleen and a fraction of liver lobe were weighed and then passed through a 70 μm cell strainer with 1 mL of lysis buffer composed of NP-40 cell lysis buffer (thermofisher), 1mM of PMSF and protease inhibitor cocktail (Sigma-Aldrich). After 30 min of ice incubation, homogenates were centrifugated for 10 min at 13000 rpm. Supernatants were collected and stored at -80°C until ELISA assay. IL-4, IL-10, IL-6, IL-17, IFN-γ and TNF-α were quantified using the ELISA Max Deluxe set (BioLegend) and the level of cytokines was adjusted according to the organ weight. The LEGENDplex bead-based immunoassay was used to quantify cytokines in blood plasma.

### Statistics

One-way ANOVA followed by the Bonferroni test was used for multigroup comparisons. A two-tailed Student’s t test for unpaired data was applied for comparisons between 2 groups (*p ≤ 0.05, **p ≤ 0.01, ***p ≤ 0.001). The non-parametric Mann-Whitney test was additionally used to confirm statistical differences between 2 groups of mice in the *in vivo* study. *p* value < 0.05 was considered to denote statistical significance. *In vitro* experiments were repeated 3 times while *in vivo* experiments were repeated at least 2 times. Data were analyzed with Prism Software (GraphPad Software Inc.). Error bars represent standard error of the mean.

## Results

### LdAg induce the expression of MHC class II on BMDCs

**Fig 1:**
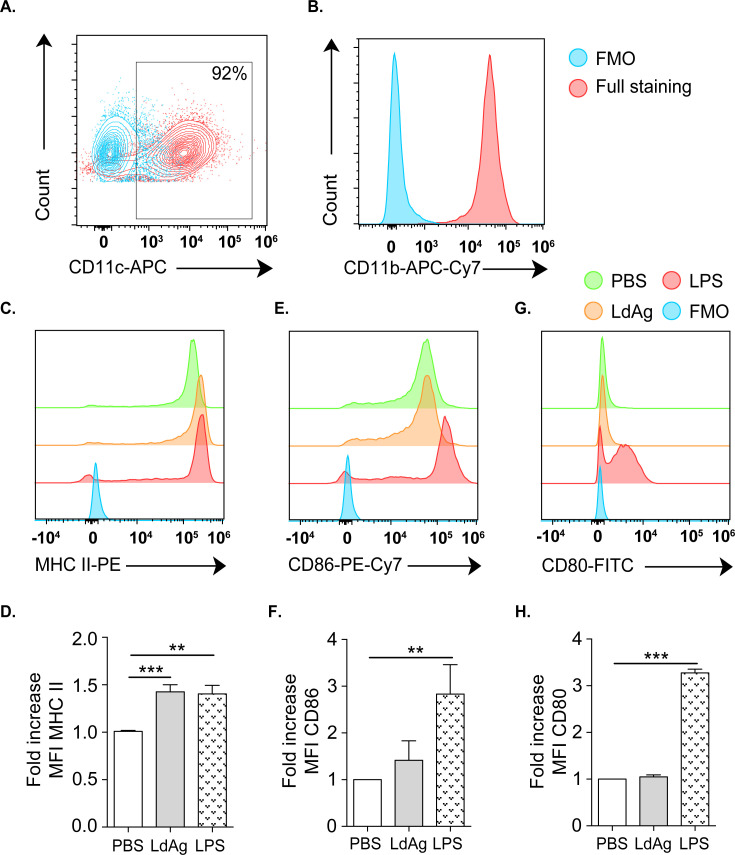
LdAg induce MHC II expression in BMDCs. (A, B) Representative flow cytometry plots showing the expression of CD11c (A) and CD11b (B) on BMDCs. (C-H) Representative flow cytometry plots showing the expression of MHC II (C), CD86 (E) and CD80 (G). Corresponding quantification are presented as mean fluorescence intensity (MFI) fold increase, calculated as the ratio between LdAg- or LPS-stimulated BMDCs compared to untreated (PBS) BMDCs (D, F, H). Data are representative of at least two independent experiments and are presented as means ± SEM (*n*  =  4; one-way ANOVA).

The efficacy of a vaccine is first determined by its capacity to induce the innate immune system. In particular, DC activation drives T cell priming and is a prerequisite for vaccine-elicited immune responses [11,30]. Therefore, we wanted to evaluate the direct recognition of LdAg by DCs that lead to their activation. For this purpose, DCs were generated from bone marrow progenitors and used as an *in vitro* cell model to analyze LdAg immunogenicity. The phenotype was then checked by flow cytometry, revealing that generated cells are mainly CD11c+ (≥90%) **(Fig 1A)** and co-express the myeloid cell marker CD11b **(Fig 1B)**. To evaluate the capacity of LdAg to induce DC activation, BMDCs were incubated with LdAg or the positive control, LPS, for 24 hours. The major histocompatibility complex class II (MHC II) and the costimulatory molecule CD86 were expectedly expressed on BMDCs without any stimulation, as revealed by the measurement of the mean fluorescence intensity (MFI). Interestingly, LdAg was as efficient as LPS in increasing the expression of MHC II (1.5-fold) **(Fig 1C and 1D)**. However, LdAg did not significantly increase the expression of CD86 and CD80 **(Fig 1E–1H)**. Taken together, our vaccine formulation has the potential to activate DCs and increase MHC II expression, suggesting the induction of antigen processing and presentation pathways.

### Intranasal and intradermal immunizations with a LdAg-based prophylactic vaccine protect mice against VL

**Fig 2:**
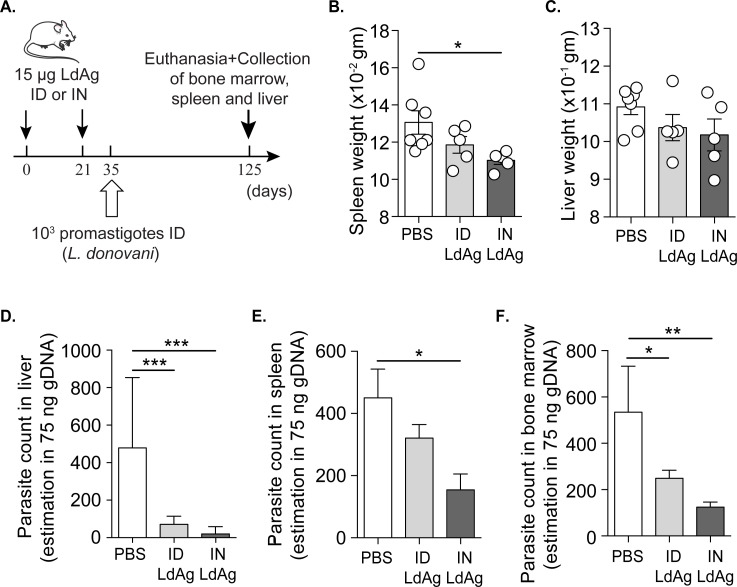
LdAg-based prophylactic vaccine decreases *Leishmania* parasitic burden. (A) Scheme representing the prophylactic vaccine protocol against VL. All mice groups were infected; PBS group represents non-vaccinated mice, ID LdAg group represents intradermally vaccinated mice and IN LdAg represents intranasally vaccinated mice. Mouse image is from smart.servier.com. (B, C) Spleen (B) and liver (C) weights measured immediately after dissection of different mice groups. (D-F) The effect of ID and IN LdAg vaccine on *L*. *donovani* parasite count in livers (D), spleens (E) and bone marrows (F) estimated by qPCR in 75 ng of tissue gDNA. Data are representative of two independent experiments and are presented as means ± SEM (*n*  =  5–7; one-way ANOVA).

To put our *in vitro* results in context and investigate the protective role of LdAg as a prophylactic vaccine, we immunized a cohort of mice with 15 μg of LdAg *via* the ID or the IN route. The boost occurred 21 days after the prime, followed by subcutaneous infection of all mice groups with *L*. *donovani* promastigotes at day 35, as described in **Fig 2A**. Infected mice were euthanized at day 125 to collect the spleen, liver and BM; all of which are known to be the primary affected internal organs in VL [31–33]. Since splenomegaly is the most common aspect of VL infection, we assessed the effect of LdAg vaccine on spleen weight. Both ID and IN LdAg immunizations led to a decreased tendency in spleen weight, but the effect was only significant with the IN route **(Fig 2B)**. The same trend was observed for liver weight, but differences were not statistically significant **(Fig 2C)**. Next, we amplified and quantified *Leishmania* genomic material using qPCR to estimate parasitic burden in the infection of the spleen, BM and liver. We observed that LdAg injection *via* both ID and IN routes dampens the parasitic burden in the liver and the bone marrow, while only the IN route significantly decreases the parasitic burden in the spleen **(Fig 2D–2F)**. Remarkably, the IN route led to a larger decrease in parasitic burden as compared to both non-vaccinated and ID-vaccinated groups. Taken together, these results suggest that LdAg-based prophylactic vaccine is efficient in the protection against VL and better protection was ensured *via* IN immunization.

### LdAg-based IN immunization induces adaptive immunity

**Fig 3:**
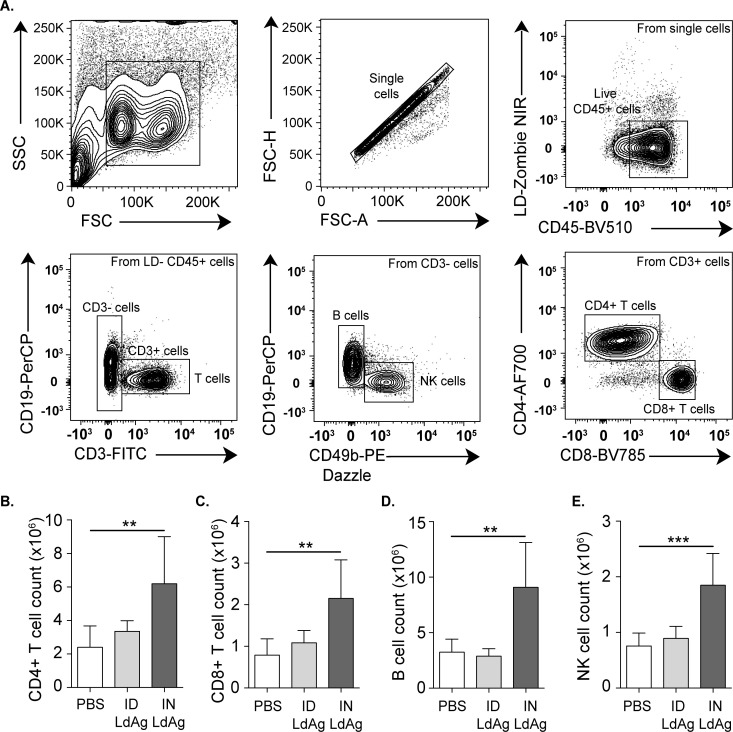
Intranasal administration of LdAg promotes lymphoid immune cells accumulation. (A) Representative flow cytometry plots providing the gating strategy for the identification of the principal immune populations in the spleen. (B-E) Impact of ID and IN LdAg immunization on the number of CD4+ T cells (B), CD8+ T cells (C), B cells (D) and NK cells (E), assessed by flow cytometry using count beads. Data are representative of two independent experiments and are presented as means ± SEM (*n*  =  5–7; one-way ANOVA).

The non-responsiveness of adaptive immune cells, including B and T cells, is associated with a poor prognostic and enhanced progression of VL infection [34]. Vaccine efficacy is closely dependent on its capacity to induce adaptive immunity in infected organs, mainly the spleen that plays a pivotal role in the control of systemic infections [35]. To assess the impact of LdAg vaccine on immune stimulation, we characterized and quantified main splenic immune cells, including B cells, T cell subsets and natural killer (NK) cells. Using flow cytometry, we identified B cells as CD45+, CD3-, CD49b-, CD19+ cells; T cell subsets as CD45+, CD19-, CD4+ for T helpers (Th) or CD8+ for cytotoxic T cells (Tc); and NK cells as CD45+, CD3-, CD19-, CD49b+ cells **(Fig 3A)**. Remarkably, LdAg immunization *via* the IN route was associated with a significant increase in the number of CD4+ T cells, CD8+ T cells, B cells and NK cells, as compared to non-vaccinated mice **(Fig 3B–3E)**. Conversely, the ID route failed to stimulate adaptive immunity, as compared to non-vaccinated and intranasally-vaccinated mice. Altogether, these data highlight the capacity of LdAg to induce potential antileishmanial response when administered *via* the IN route.

### Intranasal LdAg vaccine induces specific IFN-γ-secreting immune cells against VL

**Fig 4:**
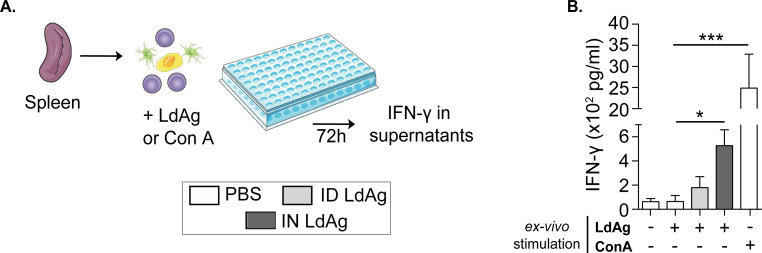
*Ex-vivo* stimulation of vaccinated mice splenocytes leads to IFN-γ secretion. (A) Spleens were processed to obtain single cell suspensions and incubated for 72 hours with LdAg or Con A to collect the supernatants. Images are from smart.servier.com. (B) Quantification of IFN-γ secretion by stimulated splenocytes *via* ELISA. Data are representative of two independent experiments and are presented as means ± SEM (*n*  =  4; one-way ANOVA).

To understand the immunological mechanisms underlying the capacity of IN LdAg vaccine to decrease the parasitic burden, we further investigated the cytokine microenvironment. *In situ* quantification showed a mixed cytokine response to the IN LdAg vaccine in spleen and liver **(S2A–S2F Fig)**. Although IFN-γ:IL-10 ratio was >1 in the spleen and ~2 in the blood **(S2G and S2H Fig)**, it is challenging to define the immune balance in such a mixed and timeline-dependent immune response [5,10]. Therefore, we next studied whether IN LdAg vaccine induces specific T cells against *L*. *donovani*. For that, we performed a lymphoproliferation test in which we stimulated splenocytes from non-vaccinated and vaccinated mice (IN and ID) with LdAg *in vitro*. We then quantified the production of IFN-γ, the main marker of cell-mediated immunity. In some conditions, splenocytes were stimulated with Con A as a positive control of activation **(Fig 4A)**. Comparisons between the different groups revealed that the *in vitro* stimulation of splenocytes from intranasally immunized mice results in remarkable and significant secretion of IFN-γ. The production of IFN-γ did not reach statistical significance in splenocytes from intradermally immunized mice as compared to non-vaccinated mice **(Fig 4B)**. This suggests that administration of the LdAg vaccine *via* the IN route ensures the efficient priming and activation of adaptive antileishmanial immunity, leading to the secretion of IFN-γ.

### Maltodextrin nanoparticles (NP) enhance LdAg capacity to activate DCs *in vitro* and induce memory T cells *in vivo*

**Fig 5:**
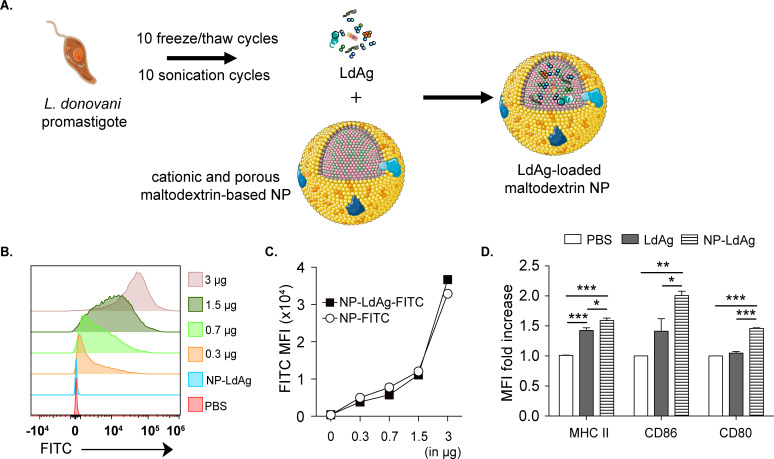
NP-LdAg formulation is uptaken by BMDCs leading to their enhanced activation. (A) LdAg were generated from *L*. *donovani* promastigotes. Cationic and porous maltodextrin-based NP were loaded with LdAg to form LdAg-loaded maltodextrin NP (NP-LdAg). Images are from smart.servier.com. (B, C) Representative cytometry plot showing the uptake of increasing concentrations of NP-LdAg-FITC (B) and FITC MFI expression in BMDCs treated with 3 μg of NP-LdAg or with increasing quantity of NP-LdAg-FITC (0.3 to 3 μg) (C). (D) MFI fold increase of MHC II, CD86 and CD80, calculated as the ratio between LdAg- or NP-LdAg-stimulated BMDCs compared to untreated (PBS) BMDCs. Data are representative of at least two independent experiments and are presented as means ± SEM (n  =  4; one-way ANOVA).

Since LdAg vaccine provides efficient protection against VL *via* the IN route, we explored the ability of delivery systems to improve the mucosal delivery and immunogenicity of the LdAg vaccine. As previously described, LdAg were prepared from *L*. *donovani* promastigotes exposed to 10 freeze/thaw cycles in alternation with 10 sonication cycles **(Fig 5A)**. In parallel, cationic and porous maltodextrin-based NP were prepared as previously described [36]. They had a size of 42 nm and a surface charge of +32 mV **(S1 Table)**. They were loaded with LdAg proteins (1:3 weight ratio) by mixing, in order to form NP-LdAg. At this ratio, all the antigens were encapsulated within the NP, as confirmed by Native PAGE **(S1 Fig)**, and no release was observed over time. This formulation was first tested *in vitro* with BMDCs. Using FITC, we investigated the capacity of BMDCs to uptake NP-LdAg. Unconjugated NP were used as a negative control. Interestingly, BMDCs incubated with increasing concentrations of NP-LdAg-FITC were associated with an increase in FITC MFI. After 24 hours of incubation with 3 μg of NP-LdAg-FITC, 100% of BMDCs were FITC positive, which was an indication of antigen uptake **(Fig 5B)**. Additionally, a similar uptake was observed with empty NP-FITC, suggesting that antigen loading does not affect these nano-sized material properties **(Fig 5C)**. To determine whether NP-LdAg formulation improves DC activation when compared to LdAg, we analyzed the expression of MHC II and CD86/CD80 costimulatory molecules. NP-LdAg were significantly efficient in MHC II induction, as well as CD86 and CD80 (1.5 to 2-fold change), that LdAg alone failed to activate **(Fig 5D)**.

**Fig 6:**
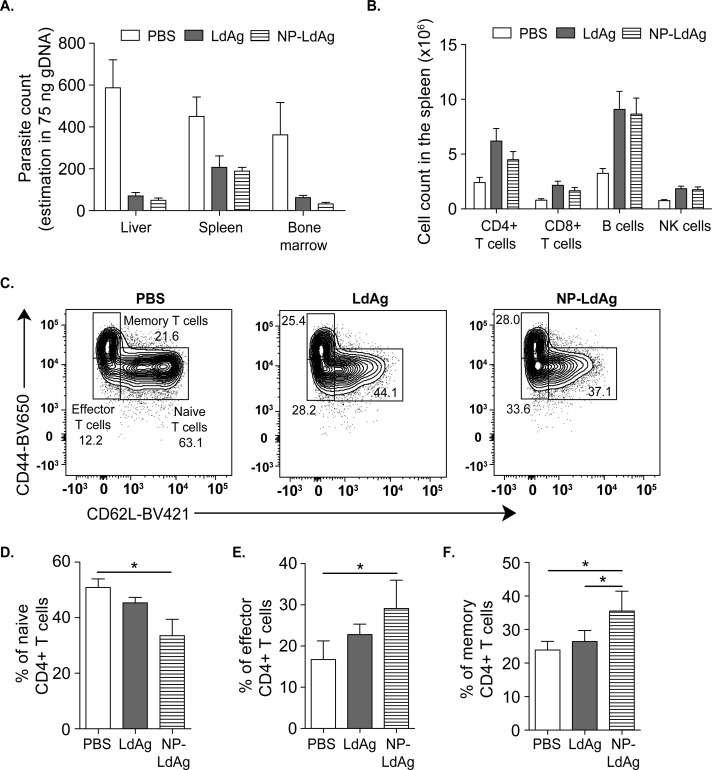
NP-LdAg formulation increases the percentage of effector and memory CD4+ T cells. (A) The effect of LdAg or NP-LdAg immunization on *L*. *donovani* parasite count in livers, spleens and bone marrows estimated by qPCR in 75 ng of tissue gDNA. (B) Impact of LdAg or NP-LdAg vaccination on the number of CD4+ T cells, CD8+ T cells, B cells and NK cells, assessed by flow cytometry using count beads. (C) Cytometry plots representing naïve, effector and memory CD4+ T cells in the spleen identified using CD62L and CD44 staining. (D-F) Percentage of naïve (D), effector (E) and memory CD4+ T cells in the spleen. Data are representative of two independent experiments and are presented as means ± SEM (*n*  =  5–7; one-way ANOVA).

Next, we evaluated the advantages of NP-LdAg formulation *in vivo* in a preventive vaccine protocol as compared to LdAg. For that, we established the same experimental procedure described in **Fig 2A** and vaccinated mice intranasally with either LdAg alone or NP-LdAg. The protective potential of NP-LdAg was comparable to LdAg in decreasing the parasitic burden in the spleen, liver and BM, as well as in inducing B cells, NK cells, CD4+ and CD8+ T cells **(Fig 6A and 6B)**. This indicates that LdAg conserve *in vivo* immunogenicity when loaded in a delivery system, with a potential modification in the immune response kinetics **(S3 Fig)**. However, NP-LdAg formulation does not significantly improve the protection against VL, as compared to LdAg in our experimental design and timeline. Given that the generation of memory T cells is one of the main features that determine the long-term effectiveness of vaccines, we compared effector and central memory CD4+ T cell generation in LdAg versus NP-LdAg intranasally-vaccinated mice. Naïve CD4+ T cells were identified as CD44lo CD62hi; effector CD4+ T cells as CD44lo CD62lo and memory CD4+ T cells as CD44hi CD62lo **(Fig 6C)**. The study of splenic CD4+ T cells revealed that NP-LdAg have a different distribution of CD4+ T cells as compared to LdAg, characterized by a significantly lower percentage of naïve CD4+ T cells and consequently higher percentages of effector CD4+ T cells and memory CD4+ T cells **(Fig 6D–6F)**. This suggests that NP-LdAg enhances DC activation and may lead to an improved induction of effector and memory Th cells as compared to LdAg. Therefore, NPs may be considered to be safe and efficient delivery systems for the future development of an intranasal vaccine against VL.

## Discussion

Visceral leishmaniasis is a poverty-related disease and is the most severe form of *Leishmania* infection. Although VL is associated with high fatality, preventive vaccines that protect susceptible people in endemic countries have yet to exist. In this study, we suggested and tested a simple and affordable vaccine made from whole *Leishmania* promastigote antigens. We demonstrated *in vivo* that this vaccine efficiently protects mice against *L*. *donovani* infection and can stimulate DCs and IFN- γ secreting cells *in vitro*.

Strong evidence supports the importance of DCs in generating a long-lasting immunity through the orchestration of the adaptive immune response [37,38]. Nowadays, IN vaccination is considered as a less invasive delivery route that is associated with more widespread immunity as compared to existing alternatives. Interestingly, IN vaccine platforms against Covid-19 are well-positioned in current clinical trials [39,40]. The efficiency of this route primarily relies on the rapid activation of antigen presenting cells, including DCs and macrophages within the nasal-associated lymphoid tissue (NALT) [41]. Several studies have shown that DCs play a pivotal role in the uptake, processing and presentation of intranasally delivered antigens to T cells in the draining lymph nodes [42–44]. Here, we performed an *in vitro* evaluation using a well-characterized DC model derived from the bone marrow [45–47]. We also demonstrated that LdAg are able to enhance the expression of MHC II but not the other maturation markers: CD80 and CD86. It is known that DC exposure to maturation stimulus inhibits MHC II ubiquitination, leading to the translocation and accumulation of peptide–MHC II complexes [48,49]. Since MHC II is a targeted pathway that reflects DC immunocompetency, newly synthetized MHC II suggests the recognition of LdAg as danger signals that lead to DC maturation.

It has been previously reported that IN vaccination with *L*. *amazonensis* or *L*. *braziliensis* antigens ensures significant protection against cutaneous leishmaniasis [17,50]. Nonetheless, the effectiveness of such a strategy against VL remains to be elucidated and the immune mechanisms are still poorly explored. In this study, we used a simple approach that utilizes brutal thermal variation followed by sonication to produce a lysate of *L*. *donovani* promastigote antigens termed LdAg. Strikingly, both ID and IN LdAg prime-boost vaccinations decrease the parasitic burden in the liver, bone marrow and spleen. However, IN delivery is significantly more protective against VL. This is consistent with the aforementioned studies demonstrating that IN vaccination decreases the parasitic burden and lesion thickness in cutaneous leishmaniasis. Therefore, IN delivery of *Leishmania* whole antigens may be a simple, cost-effective, and non-invasive strategy that should be reconsidered in the development of vaccines against both visceral and cutaneous leishmaniasis.

Immuno-proteomic techniques are now extensively used to define immunogenic candidates in both promastigote and amastigote antigens [51–53]. Based on several previous studies highlighting the presence of highly immunogenic antigens in the promastigote form that initiates infection [54–56], we designed our prophylactic vaccine with total antigens from promastigote and identified the optimal mode of administration. Interestingly, no liver and spleen biopsies from IN LdAg vaccinated mice yielded viable parasites *in vitro*, suggesting significant protection against promastigote infection. Since the induced immune protection may be limited to the promastigote form, we intend to strengthen our prophylactic vaccine with total antigens of axenic amastigote, which is the invasive form in the mammalian host. Therefore, further studies should be determinant of vaccine effectiveness in different parasite stages. Additionally, more clinically relevant animal models, including the hamster, may pave the way for translational research and clinical trials.

The immune response in VL infection is very complex and implicates various mechanisms that prevent efficient eradication of the parasite [10]. In particular, promising vaccines against VL may be capable of reverting the immune balance towards a Th1/Th17 response. In line with other studies that have shown the potential of IN vaccines’ ability to induce specific cellular-mediated anti-infection responses [57–62], our prime-boost IN immunization resulted in IFN-γ secretion after *in vitro* restimulation of splenocytes. This is reflective of a specific antileishmanial response in the spleen avoiding the persistence of a chronic infection [5]. In accordance with these *in vitro* results, we observed a remarkable increase in the number of the top IFN-γ secreting cell candidates, T and NK cells. This was also correlated with a significant decrease in the parasitic burden with IN, but not with ID vaccination. It is worth mentioning that VL in mice is characterized by an organ restricted immunity, in which failure of splenic immunity is responsible for the outbreak of *Leishmania* parasites in the different visceral organs [31,63]. Understanding the immune mechanisms associated with LdAg-IN immunization confirms a positive correlation between the immune response and the efficient protection against VL.

Since the majority of soluble antigens are largely not taken up by antigen presenting cells (APCs), growing interest in delivery systems has increased in the last decades. In particular, nano-antigenic formulations provide adequate delivery systems in infectious diseases, ameliorating the antigen stability, delivery and immunogenicity [64,65]. Interestingly, a recent study has also demonstrated the safety and effectiveness of nano-encapsulated retinoic acid as an adjuvant for IN vaccination against cutaneous Leishmaniasis [66]. In parallel, we have established a stable and adjuvant-free vaccination approach based on the use of maltodextrin nanoparticles [27,67]. This technology has led to the development of a vaccine against Toxoplasmosis that has been validated in mouse and sheep [68–70]. Here we evaluated the proof of concept of the same technology in a nanoparticle platform loaded with *L*. *donovani* promastigote antigens for IN delivery. Interestingly, this NP formulation acts as safe delivery system that increases LdAg immunogenicity *in vitro*, since NP-LdAg formulation improves the expression of the costimulatory molecules CD80 and CD86 on DCs, as well as MHC II; all of which are necessary for immune synapse formation and T cell priming. In our experimental conditions and short timeline, NP-LdAg formulation did not improve the protective capacity of LdAg against VL. Nevertheless, the remarkable increase in the percentage of effector and memory CD4+ T cells is very promising in the development of antileishmanial vaccine [71,72]. Indeed, meaningful immune memory response is a prerequisite for long-lasting immunity and is the outcome of naïve T cell priming in response to antigenic peptides complexed to MHC on DCs.

In conclusion, LdAg vaccine ensures protection against VL largely through the IN route, and to a lesser extent via the ID route. Consequently, our complete prophylactic vaccine strategy, relying on the IN immunization with total antigens from *L*. *donovani* promastigotes is successful in conferring an efficient immune protection against VL. Nevertheless, many limitations should be taken into consideration in this study, including the lack of translational clinical data and restricted investigation of long-lasting immunity. Therefore, it is anticipated that greater insight into this promising strategy can be further evaluated and completed in future fundamental and translational studies.

## Supporting information

S1 Table Characterization of NP size and charge. The size of the nanoparticles (NP) was measured by dynamic light scattering (DLS) and is expressed as Z-average (average particle size) and Number (most abundant size), in nm. The NP surface charge was measured by electrophoretic light scattering (ELS) and is expressed in mV. PDI: polydispersity index. All these measurements were performed in water and at room temperature.
(EPS)

S1 Fig Characterization of NP-LdAg formulation on Native PAGE gel. 3 μg of LdAg alone (LdAg well) or mixed with 9 μg NP (NP-LdAg well, 1/3 weight ratio), and 9 μg NP alone (NP) were deposited onto native polyacrylamide gel. After a silver nitrate staining, the percentage of encapsulation was measured using Image J software. The absence of protein in the NP-LdAg well confirms their complete association in the NP.
(EPS)

S2 Fig *In situ* quantification of relevant cytokines following intranasal LdAg vaccine. (A-H) Spleens and livers were collected and lysed using the NP-40 buffer. *In situ* levels of IFN-γ, IL-6, TNF-α, IL-17, IL-4 and IL-10 were assessed in the resulting homogenates using ELISA. Cytokine concentrations were adjusted per gram of organ (n  = 5). Levels of IFN-γ, TNF-α, IL-2, IL-6, IL-4, IL-10 and IL-17 were assessed in blood plasma using LEGENDplex bead-based immunoassay (*n*  =  5–7).
(EPS)

S3 Fig *In situ* quantification of relevant cytokines following intranasal NP-LdAg vaccine. (A-F) Spleens and livers were collected and lysed using the NP-40 buffer. *In situ* levels of IFN-γ, IL-6, TNF-α, IL-17, IL-4 and IL-10 were assessed in the resulting homogenates using ELISA. Cytokine concentrations were adjusted per gram of organ (n  =  5).
(EPS)
